# Cardiovascular adverse events in pregnancy: A global perspective

**DOI:** 10.21542/gcsp.2021.5

**Published:** 2021-04-30

**Authors:** Susy Kotit, Magdi Yacoub

**Affiliations:** 1 Aswan Heart Centre, Aswan, Egypt

## Abstract

Pregnant women with heart disease are vulnerable to many adverse cardiovascular events (AE). AEs during and after pregnancy continue to be important causes of maternal mortality and morbidity worldwide, with huge variations in burden in different countries and regions. These AEs are classified as having direct or indirect causes, depending on whether they are directly caused by pregnancy or due to some pre-existing disease and/or non-obstetric cause, respectively.

The risks continue throughout pregnancy and even after childbirth. Apart from immediate complications during pregnancy, there is increasing evidence of a significant link between several events and the risk of cardiovascular disease (CVD) later in life.

A significant number of pregnancy-related deaths caused by cardiovascular disease are preventable. This prevention can be realized through increasing awareness of cardiovascular AE in pregnancy, coupled with the application of strategies for prevention and treatment. Knowledge of the risks associated with CVD and pregnancy is of extreme importance in that regard.

We discuss the global distribution of cardiovascular maternal mortality, adverse events during and after pregnancy, their predictors and risk stratification. In addition, we enumerate possible solutions, particularly the role of cardio-obstetric clinics.

## Introduction

Pregnant women with heart disease have pronounced vulnerability to adverse cardiovascular events. Diagnosis and treatment of heart disease in pregnancy is difficult due to similarities between disease manifestations and normal physiological changes.

Cardiovascular adverse events (AEs), during and after pregnancy continue to be important causes of maternal mortality and morbidity worldwide, with huge variations in burden in different countries and regions.^1,2^ These AEs are classified as having direct or indirect causes, depending on whether they are directly caused by pregnancy or due to some pre-existing disease and/or non-obstetric cause, respectively.^3–5^

Cardiovascular disease is the single largest cause of indirect maternal mortality,^3,6^ accounting for over 33% of pregnancy-related maternal deaths.^6–11^ Additionally, maternal heart disease complicates up to 4% of pregnancies^12–14^ and up to 16% of pregnancies in women with previous cardiac conditions,^13^ with risk depending on the underlying cardiac condition.^13,15–17^

Over 50% of maternal deaths occur post-partum.^18^ Late maternal mortality is defined as death more than 42 days (and up to one year) after child birth.^19^ Importantly, the currently cited figures almost certainly constitute an underestimate.^19^

It is estimated that up to 68% pregnancy-related deaths caused by cardiovascular conditions are preventable.^6,20^ This can be achieved, through increasing awareness, coupled with applying strategies for prevention and treatment. Knowledge of the risks associated with CVD and pregnancy is of extreme importance in that regard.

We discuss the global distribution of cardiovascular maternal mortality, the adverse events during and after pregnancy, their predictors and risk stratification. In addition, we enumerate possible solutions, particularly the role of cardio-obstetric clinics.

### Incidence and global distribution of maternal mortality

According to the World Health Organization (WHO)^21^ and Global Burden of Disease (GBD),^1,2^ in 2017 there were up to 295,000 maternal deaths globally, leading to an estimated global maternal mortality rate (MMR) of 211 per 100,000 live births.

**Figure 1.**
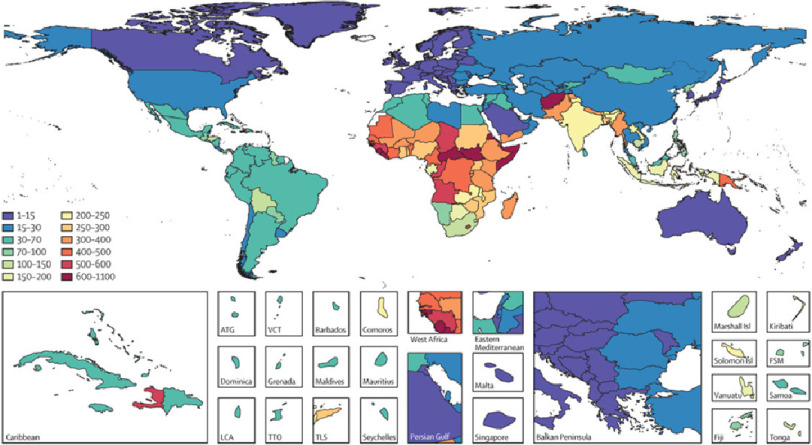
Maternal mortality ratio (MMR; number of deaths per 100,000 livebirths) for countries and territories, GBD 2015.^23^

In 2017, every day approximately 810 women died from preventable causes related to pregnancy and childbirth, with 94% of all maternal deaths occurring in low and middle-income countries (LMIC’s).^22^ Sub-Saharan Africa alone accounted for roughly two-thirds of the estimated global maternal deaths.^21^ In Egypt the number of maternal deaths was 1,316 in 2017 with a MMR of 62 per 100,000 livebirths.^8,23^ The regional distribution and pattern of maternal death reported by the GBD varies considerably around the world.^23–25^ (Figure 1).

### Causes of maternal mortality and burden of disease

**Figure 2.**
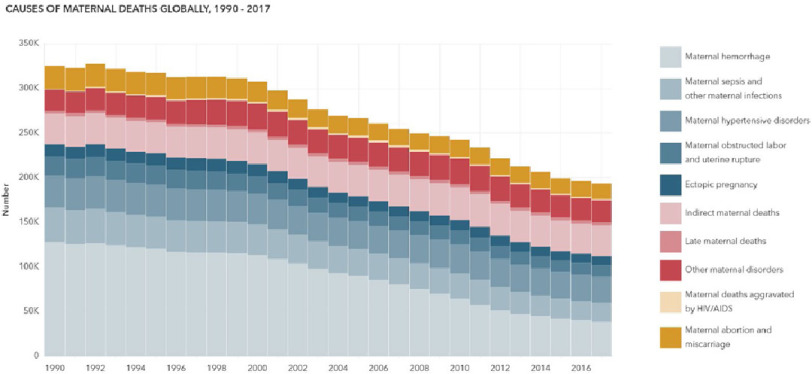
Causes of maternal deaths globally, 1990–2017.^1,2^ (A) maternal hypertensive disorders: High blood pressure during pregnancy in women who did not already have hypertension, or preeclampsia in women with preexisting hypertension. (B) Indirect maternal deaths: Deaths due to preexisting conditions made worse by physiologic effects of pregnancy. (C) Late maternal deaths: Deaths due to any cause that occurs six weeks to 12 months after pregnancy. (D) Other maternal disorders: All other direct maternal disorders, including anemia in pregnancy, gestational diabetes and embolism.

Up to 1990, direct causes accounted for over two-thirds of maternal mortality worldwide. There has been a significant decrease in overall maternal mortality over the years due to progressive drops in direct causes, however, the contribution of indirect and late maternal deaths, as well as maternal hypertensive disorders, has remained unchanged (Figure 2).^1,2^

### Regional and national variation in incidence and causes of maternal mortality

**Figure 3.**
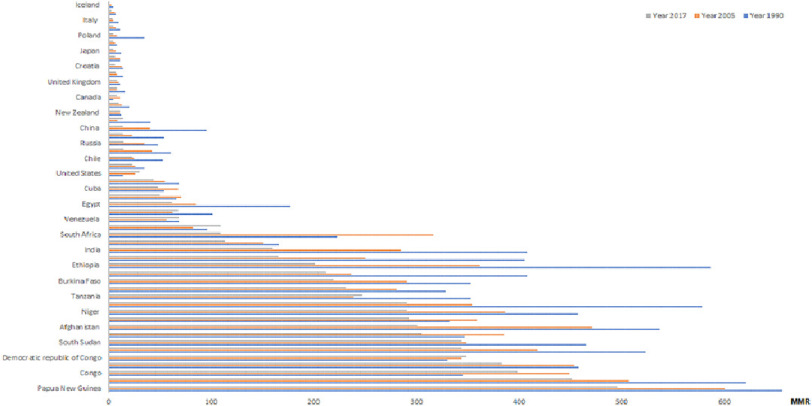
MMR per country across 1990, 2005 and 2017.^1,2^

According to the GBD, maternal mortality rate (MMR) varied from 1 to 496 per 100,000 live births (Figure 3) in 2017.^1,2^ The alarmingly high incidence in MMR in some countries continues, with very few exceptions.

The regional incidence and cause of maternal mortality varies considerably from one country to another (Table 1). In addition, the representation of maternal hypertensive disorders, indirect maternal deaths as well as the reported late maternal mortality also differs across countries.

**Table 1:** Global distribution in incidence, causes and timming of maternal mortality by MMR (2017).^1,2^ The reported late maternal deaths represented 21.9% of total maternal mortality in Chile, compated to 0.08% in Iceland.

**Country**	**Number of maternal deaths (2017)**	**MMR (2017)**	**Top mortality disorder**	**Maternal hypertensive disorders(% total maternal deaths)**	**Indirect maternal deaths(% total maternal deaths)**	**Late maternal deaths(% total maternal deaths)**
**Globally**	193,639	140	Other maternal disorders	15.17	17.61	1.74
**Iceland**	0	1	Other maternal disorders	0.08	0.03	0.08
**Sweden**	4	3	Other maternal disorders	7.25	2.50	7.50
**Italy**	17	4	Other maternal disorders	7.82	1.29	1.65
**Spain**	20	5	Other maternal disorders	11.40	1.20	1.50
**Poland**	16	5	Other maternal disorders	6.31	0.88	6.50
**Australia**	15	5	Other maternal disorders	5.07	1.53	9.07
**Japan**	46	5	Other maternal disorders	7.87	9.93	3.50
**Netherlands**	11	6	Other maternal disorders	11.09	2.36	2.91
**Croatia**	2	6	Other maternal disorders	5.50	7.00	8.00
**France**	52	7	Other maternal disorders	11.27	9.54	2.54
**United Kingdom**	62	8	Other maternal disorders	9.11	14.56	6.27
**Portugal**	7	8	Other maternal disorders	11.14	4.29	0.86
**Canada**	31	8	Other maternal disorders	6.10	12.26	16.94
**South Korea**	42	10	Other maternal disorders	10.24	6.98	2.29
**New Zealand**	6	11	Other maternal disorders	1.33	37.00	12.17
**Latvia**	3	14	Other maternal disorders	1.67	1.67	5.33
**China**	2241	14	Other maternal disorders	14.80	6.80	0.98
**Vietnam**	213	14	Maternal hemorrhage	14.63	13.50	2.77
**Russia**	250	15	Other maternal disorders	11.33	12.35	2.08
**Kazakhstan**	53	15	Other maternal disorders	8.32	6.53	7.66
**Chile**	56	23	Late maternal death	17.27	19.84	21.91
**Iran**	298	23	Indirect maternal disease	10.58	33.20	9.95
**United States**	1171	30	Other maternal disorders	10.12	15.65	17.12
**Mexico**	1120	44	Maternal hemorrhage	23.45	19.17	7.22
**Cuba**	53	48	Indirect maternal disease	7.09	21.43	17.06
**Colombia**	427	50	Indirect maternal disease	23.16	24.17	11.71
**Egypt**	1316	62	Other maternal disorders	15.76	19.68	3.67
**Brazil**	2,054	68	Maternal hypertensive disorders	20.93	18.23	6.23
**Venezuela**	395	69	Maternal hemorrhage	26.26	22.81	2.83
**Dominican Republic**	237	109	Hypertensive disorders	26.21	17.34	9.92
**South Africa**	1191	109	Indirect maternal disease	15.44	35.03	1.89
**Uganda**	1753	113	Indirect maternal disease	15.22	22.27	0.72
**India**	39,428	160	Other maternal disorders	11.50	13.16	1.78
**Indonesia**	6627	165	Maternal hemorrhage	26.69	2.19	1.41
**Ethiopia**	7451	201	Maternal hemorrhage	15.56	17.38	0.73
**Mozambique**	2090	212	Indirect maternal disease	15.07	25.13	5.29
**Burkina Faso**	1863	219	Indirect maternal disease	12.40	28.46	0.74
**Nigeria**	17982	231	Maternal hemorrhage	5.22	16.79	1.68
**Tanzania**	4916	247	Maternal hypertensive disorders	24.61	21.65	0.79
**Mali**	2559	291	Indirect maternal disease	6.63	31.18	1.23
**Niger**	2930	291	Maternal sepsis and other maternal infections	2.00	26.25	0.87
**Kenya**	3990	292	Indirect maternal disease	15.70	23.88	1.19
**Afghanistan**	4095	301	Indirect maternal disease	9.11	38.79	0.66
**Cote d’Ivoire**	2631	305	Indirect maternal disease	9.47	28.27	0.67
**South Sudan**	1410	344	Maternal hemorrhage	5.95	8.82	0.62
**Somalia**	2363	344	Maternal hemorrhage	9.10	16.98	0.56
**Democratic republic of Congo**	10166	349	Indirect maternal disease	10.99	26.59	0.68
**Chad**	2745	383	Maternal hemorrhage	6.29	14.41	0.78
**Congo**	522	398	Indirect maternal disease	11.60	26.63	0.80
**Guinea**	1916	451	Maternal hemorrhage	8.35	25.25	0.82
**Papua New Guinea**	1532	496	Indirect maternal disease	8.86	34.92	0.59

### Details of adverse cardiovascular events

#### Direct maternal adverse cardiovascular events

##### Hypertensive disorders of pregnancy.

**Figure 4.**
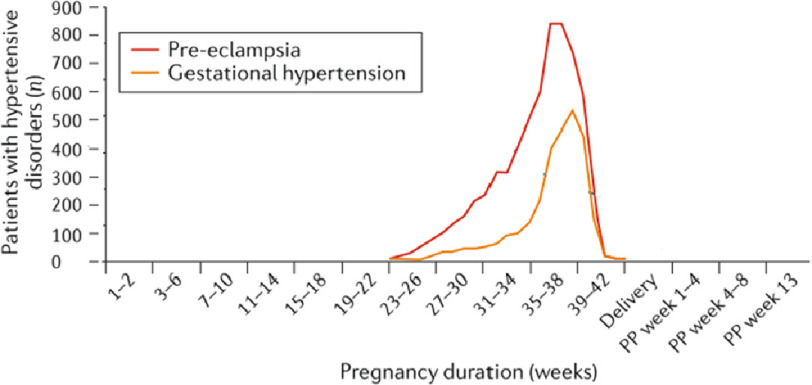
Onset of hypertensive disorders at different stages of pregnancy and postpartum (PP) among women without chronic hypertension (adapted from Ramlakhan et al).^41,43^

Chronic hypertension, gestational hypertension and preeclampsia^26^ are important causes of maternal and perinatal morbidity and mortality,^9,27–31^ particularly toward the end of pregnancy (Figure 4). Novel diagnostic methods and therapies have recently been reviewed.^32^

Gestational hypertension is defined as new-onset hypertension arising after 20 weeks of gestation^33,34^ and occurs in 10% of women.^35^ It is associated with acute and chronic cardiovascular changes leading to an increased risk for hypertension throughout life,^36,37^ and a 4-fold increased risk of future maternal cardiovascular events.^37–40^

#### Indirect maternal adverse cardiovascular events

Cardiovascular disease is the single largest cause of indirect maternal mortality.^3,6^ Patients who experience complications during pregnancy may also be at higher risk of cardiac events later in life.^44^

### Arrythmias

The altered cardiac anatomy during pregnancy can elicit new onset arrhythmia or prompt the recurrence of preexisting arrhythmias.^45,46^ An increased incidence of cardiac arrhythmias has been reported during pregnancy^47^ in patients with and without identifiable heart disease. Arrhythmias are responsible for complications in 67 per 100,000 pregnancies,^49^ mainly in the form of atrial fibrillation (27 per 100,000 pregnancies) and supraventricular tachycardia (22 per 100,000 pregnancies).^48–50^

### Heart failure

**Figure 5.**
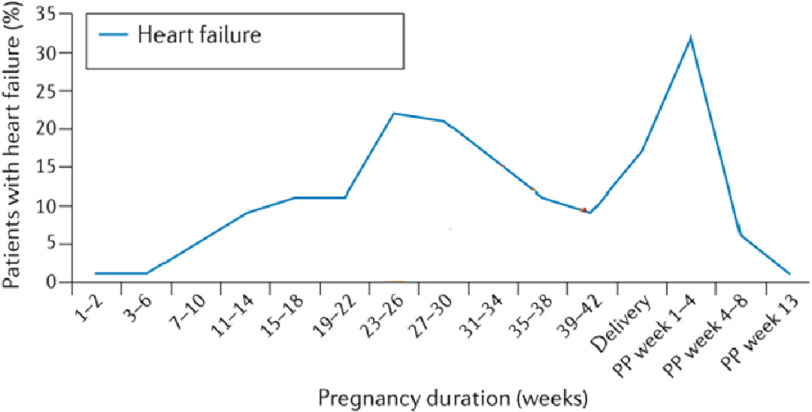
Timing of heart failure in women with structural heart disease at different stages of pregnancy and postpartum (PP) (adapted from Ramlakhan et al).^41,42^

Heart failure (HF) remains the most common complication during pregnancy among all women with heart disease, regardless of the cardiac pathology.^42,51^ Heart failure can occur during, or immediately after pregnancy (Figure 5). Despite the poor prognosis associated with the diagnosis of HF during pregnancy, and the fact that prevalence of HF among pregnant women has increased over the years - particularly in the post-partum period,^52^ data in the literature are scarce.

### Peri-partum cardiomyopathy

**Figure 6.**
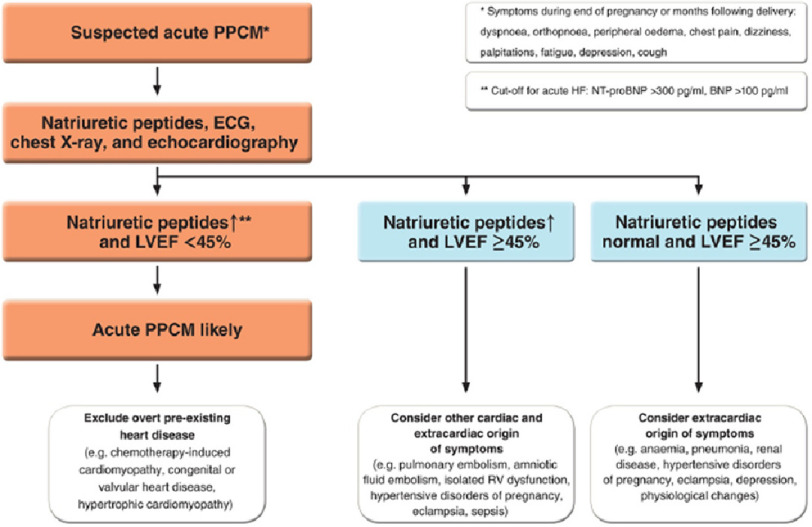
Diagnostic pathway in patients with suspected peripartum cardiomyopathy (PPCM). BNP, B-type natriuretic peptide; ECG, electrocardiogram; HF, heart failure; LVEF, left ventricular ejection fraction; NT-proBNP, N-terminal pro-B-type natriuretic peptide; RV, right ventricular.^60^

Peri-partum cardiomyopathy (PPCM) is an idiopathic cardiomyopathy presenting with heart failure secondary to left ventricle systolic dysfunction towards the end of pregnancy or in the months following child birth.^53–56^ At diagnosis, the majority of patients have severe symptoms (NYHA III/IV) and LVEF < 35%, with regional variations in presentation and outcomes^57–59^ (Figure 6).

**Figure 7.**
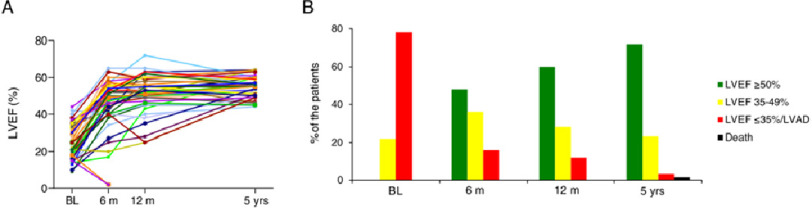
Time course of left ventricular function (adapted from.^68^) (A) Changes in left ventricular ejection fraction (LVEF) from baseline (BL) to 5-year follow-up. Remarkably, LVEF further improves even after 1 year. (B) Proportion of patients with full cardiac recovery constantly increases. At 5-year follow-up, 72% had recovered completely and 23% partially. No recovery was observed in 5%. Death occurs up to 5 years.

The incidence of PPCM varies markedly from 1–100 per 10,000 live births depending on the region.^59,61–63^ PPCM has the highest mortality rate in pregnancy, with a worldwide mortality of 2.4%,^57^ although it may be underdiagnosed. PPCM leads to substantial maternal and neonatal morbidity and mortality, with less than half of all cases recovering full cardiac function^58,64–67^ (Figure 7). Six-month mortality is around 6%, mainly due to heart failure.^58^ Neonatal death is around 5%, although with marked regional variation.^63^

Management follows the guidelines of HF. In non-responsive patients, other pharmacological agents - such as bromocriptine and prolactin-blocking therapy with dopamine D2 receptor agonists - have been tried with variable results.^68^

Contributing factors to PPCM include genetic predisposition^69^ as well as auto-immune responses.^70–72^ Using such factors, all be it indirectly, to define phenoclusters^73^ - it could be possible to identify novel therapeutic targets to guide personalized medicine in PPCM.

For a small proportion of patients with rapidly-progressive PPCM resistant to conventional therapy, the use of the current generation of left ventricular assist devices can give long-lasting “cures” in some patients (personal experience in Harefield and Aswan series).^74,75^

Registries on the condition will provide fundamental data on predisposing factors, potential aetiologia and regional variations.^57,63,76,77^

### Mechanical valve thrombosis

Even with the right care, the incidence of thromboembolism during pregnancy is estimated from 7–23% with half of these cases being mechanical valve thrombosis (MVT),^78^ which is associated with 20% mortality.^79^ Thrombosis is the most life-threatening complication for women with prosthetic heart valve, during pregnancy.^78,80^ The chance of a successful uncomplicated pregnancy, which depends on the balance between the thrombotic and bleeding risks, is around 57%.

### Acute coronary syndromes

The incidence of coronary artery disease (CAD) in women of childbearing age is unclear and varies between countries.^81,82^ CAD, is a major cause of maternal death and accounts for over 20% of maternal cardiac deaths,^83^ especially in the form of acute coronary syndromes (ACSs).^84^ The estimated incidence of 6.2 per 100,000 deliveries^85–88^ nearly 4 times higher than in non-pregnant women^89^ and reflects the growing prevalence of cardiovascular risk factors in the pregnant population.^90^

### Risk stratification

**Figure 8.**
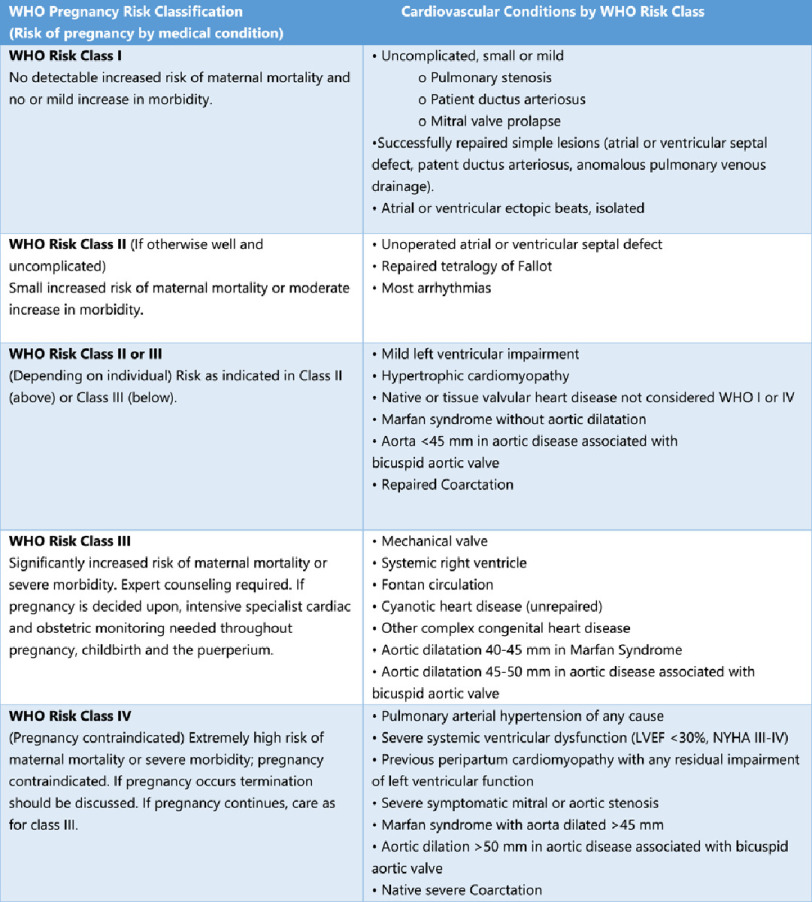
Modified WHO classification of maternal cardiovascular risk: application.^92^

Several tools have been developed to estimate morbidity and mortality risk in pregnant women with cardiac disease, such as CARPREG^13^ and ZAHARA.^12^ However, the best estimate of the risk of cardiovascular events during pregnancy in pre-existing heart disease is the WHO’s^91^ (Figure 8), as it integrates congenital and acquired heart disease.

**Figure 9.**
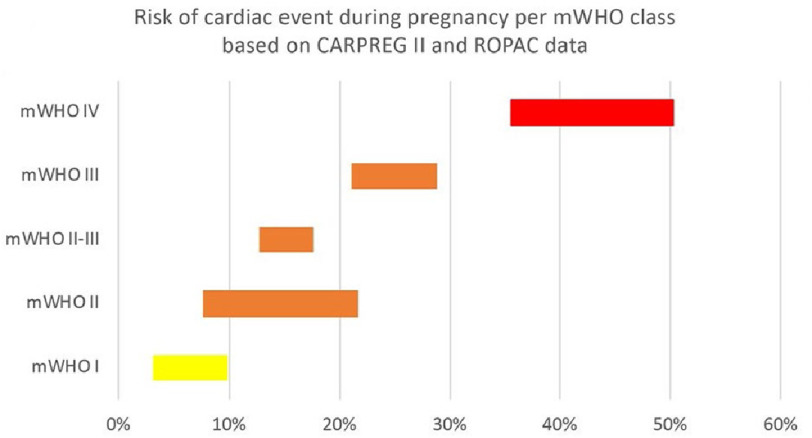
Risk of cardiac event during pregnancy per mWHO class based on CARPREG II and ROPAC data. 12,13,93 adapted from.^94^

The risk of cardiac adverse events during pregnancy are significantly increased in mWHO IV compared to mWHO I (Figure 9).

Based on the modified WHO risk classification for milder conditions, where the risk of pregnancy is very low to moderate, the needed care might be limited to a few visits during pregnancy, while in case of high risk of complications, a more frequent follow-up schedule is recommended. Women in the highest risk group (WHO IV) should be advised against pregnancy. In case of pregnancy, strict monitoring is required.^92^ More comparative studies should be performed in order to define the most accurate risk index for pregnant women with heart disease.^91,95^

### The role of comprehensive cardio-obstetric clinics

**Figure 10.**
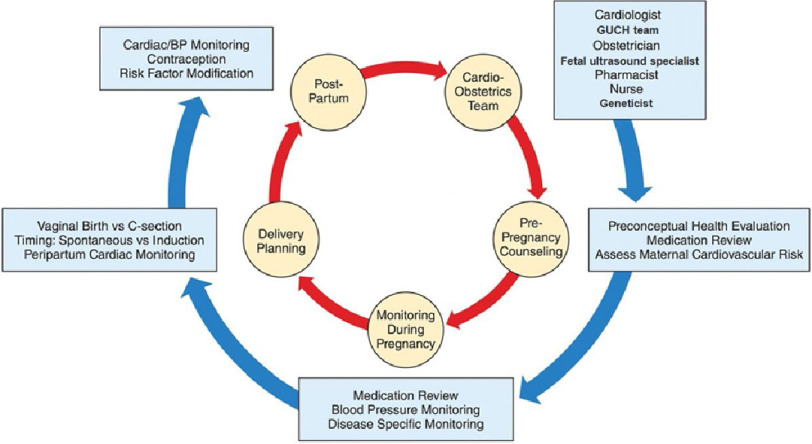
Cardio-obstetrics team in the management of women before pregnancy, during pregnancy, and postpartum (GUCH: grown-up congenital heart disease) Adapted from.^115^

The concept of multidisciplinary cardio-obstetric clinics (Figure 10) has evolved and been applied in several countries, with extremely encouraging results^96–114^ and excellent survival rates of mothers even with complex diseases, and their offspring.^24^

However, the need for cardio-obstetric clinics remains un-met, with most programs to be found in developed countries and with only a few centers continuing follow-up long term.^24,101,102^

Although these initiatives might be feasible in large metropolitan areas, smaller towns, rural communities and remote regions are completely neglected. There is an urgent need for action and worldwide implementation of cardio-obstetric clinics, strategically placed, in order to reach the majority of those in need.

Integrated, tailored and dynamic healthcare services responding to current state of disease burden and initiatives are essential. Cardio-obstetric care clinics (COcare) have been initiated in the metropolitan Aswan Heart Center as well as its rural branch in Ballana.^116^

## Conclusions

Preventable maternal cardiovascular adverse events continue to be a global problem with an unacceptably high burden of disease. This requires urgent concerted efforts from governments, individuals and professionals, who have first-hand experience of the magnitude of the problem. Multidisciplinary cardio-obstetric care should also play an important role.
